# The Hidden Power of Black Pepper: Exploring Piperine’s Role in Cancer

**DOI:** 10.1007/s11130-025-01374-z

**Published:** 2025-05-29

**Authors:** Ezgi Nur Cinar, Nevin Sanlier

**Affiliations:** Department of Nutrition and Dietetics, School of Health Sciences, Ankara Medipol University, 06050 Altındag, Ankara, Turkey

**Keywords:** Anticancer, Black pepper, Natural therapeutic agents, Piperine, *Piper nigrum* L

## Abstract

Cancer is a multifaceted disease that occurs when cells proliferate and migrate in an uncontrolled and unregulated manner. The development of cancer is the result of the interaction of a number of factors, including genetic mutations, environmental factors and lifestyle habits. There are many pharmacological and natural compounds that can be used to prevent and/or treat cancer. Piperine, a naturally occurring compound with multiple therapeutic properties, is the primary bioactive component of black pepper (*Piper nigrum* L.), a member of the *Piperaceae* plant family. In recent years, it has attracted much interest as a potentially useful agent for the preventive and curative management of cancer. Results from studies of human cancer cell lines and advanced animal tumour models suggest that there are multiple pathways by which piperine may affect cancer development and metastasis. This review examines the molecular and cellular mechanisms through which piperine exerts its effects on cancer formation and progression, as well as its potential effects on various types of cancer.

## Introduction

Cancers constitute a family of diseases defined by the uncontrolled proliferation of cells, resulting from the loss of normal control over the processes of growth and division [1]. Cancer is now a significant public health concern, accounting for nearly one in six of all deaths (16.8%) and one in four deaths (22.8%) from non communicable diseases globally [2]. It is further estimated that 20 million new cases of cancer and 9.7 million cancer associated deaths occurred globally in 2022 [3]. In the last few years, a range of alternative strategies have been the subject of investigation in the context of cancer treatment, in addition to the established approaches of surgery, radiotherapy, and chemotherapy [4]. It is widely recognised that lifestyle factors such as avoiding smoking, maintaining a healthy weight, engaging in regular physical activity, limiting alcohol consumption and adhering to a nutritious diet can significantly reduce the risk of developing several types of cancer, particularly those strongly associated with modifiable behaviors such as lung, colorectal and breast cancer [5]. Moreover, the combination of specific phytochemicals, such as polyphenols and alkaloids extracted from medicinal plants with healthy nutritional patterns has shown potential in cancer prevention and adjunct therapy. These compounds are attracting attention due to their capacity to reduce cell proliferation, induce apoptosis, delay metastasis, and inhibit angiogenesis while exhibiting relatively low toxicity profiles [6].

Black pepper (*Piper nigrum* L.), historically referred to as the ‘king of spices’, is a member of the family *Piperaceae* and one of the most widely used spices globally. Black pepper is widely used in traditional medicine for its therapeutic properties in the treatment of many health problems such as headaches, rheumatic diseases, or sore throat and flu, as well as for improving blood circulation [7]. It contains piperine (1–piperoylpiperidine), an alkaloid responsible for its unique aroma and pungent taste, which has many potential health benefits [8]. Piperine, regarded as the principal bioactive constituent of black pepper, is documented to be most abundant in black pepper (*Piper nigrum* L., 9%), while it is also present at intermediate levels in long pepper (*P. longum* L., 4%) and Balinese pepper (*Piper retrofractum* Vahl, 4.5%) [9]. While the outer layer of pepper seeds accounts for 7–8% of the total piperine content, the inner core contains more than 90%. Consequently, the core of black pepper, designated as white pepper, is the primary resource for piperine extraction [10].

Piperine has been demonstrated to exhibit a multitude of therapeutic benefits, exerting anti–inflammatory, immunosuppressive, antimicrobial, antihypertensive, antioxidant, antidiabetic, analgesic, and antidepressant effects [11, 12]. Piperine has emerged as a promising anticancer agent, either alone or in combination with conventional chemotherapeutics. It has been shown to regulate several key signalling pathways involved in cancer development, including the STAT-3, NF–κB, PI3 K/AKT, and JNK/p38–MAPK pathways [13]. Researchers have proposed that piperine may possess the capacity to impede cell proliferation and migration in vitro at certain doses (75- 200 µM) and durations of incubation (24–48 h), cause cell cycle arrest, stimulate apoptosis, regulate redox homeostasis, and modulate angiogenesis in malignant cells [14]. Despite the large evidence supporting the therapeutic potential of piperine, its application in clinical oncology remains limited, mainly due to pharmacokinetic constraints and lack of mechanistic insights [15]. The objective of this review is to examine piperine’s efficacious mechanisms of action against the onset and advancement of diverse types of cancer at the molecular and cellular levels.

## Study Design

For this review, studies investigating the effects of piperine on cancer development and progression were identified and examined based on the current scientific literature. A comprehensive literature review was carried out using a range of databases including PubMed, Web of Science, and Google Scholar with key terms including ‘piperine’, ‘*Piper nigrum*’, ‘black pepper’, ‘cancer and phytochemicals’, and ‘cancer and antioxidants’. A search was conducted for peer – reviewed articles that are in English and focus on the molecular and cellular mechanisms by which piperine exerts effects on different cancer types. The titles alongside abstracts from the acquired articles underwent a process of scanning and assessment for their appropriateness. Subsequently, data regarding the study designs employed, the specific cancer types investigated, the methodologies applied, and the resultant findings from the chosen studies were gathered and subjected to analysis.

## Chemical Structure and Derivatives of Piperine

Piperine (1-[5-[1,3-benzodioxol-5-yl]−1-oxo-2,4-pentadienyl]piperidine), a nitrogen-containing alkaloid molecule, was first isolated in the form of a yellow crystalline solid by the Danish chemist Hans Christian Orstedt in 1820, from the dried fruit extract of pepper [15]. Chemically, piperine molecule has a piperidine ring conjugated to a methylenedioxyphenyl moiety via an α,β-unsaturated carbonyl system. This rigid, planar structure contributes to its pronounced lipophilicity and low aqueous solubility (40 mg/L at 18 °C), which significantly inhibits its oral bioavailability and therapeutic use [8]. To overcome these challenges and optimize its pharmacological potential, several structural modifications have been made to develop more potent and bioavailable derivatives [16]. A recent study demonstrated that piperine derivative HJJ_3_5 significantly suppressed cell proliferation, migration and invasion in HCT116 colorectal cancer cell line via SNAI1-mediated EMT compared to piperine [17]. Another study showed that a novel piperine derivative YL-1–9 exhibited significant antitumor effects against breast cancer by inhibiting cell proliferation, migration and invasion, as well as inducing cell cycle arrest and apoptosis. These effects were mediated by modulation of CDK4/6-cyclin D-Rb-E2 F and Caspase 3/Bax/Bcl-2 signaling pathways, highlighting YL-1–9 as a promising therapeutic candidate [18]. Similarly, another novel synthesized derivative MHJ-LN also showed potent anticancer activity in triple negative breast cancer models by activating the MDM2-p53 pathway and inducing G1/S phase arrest [19]. Besides structural modifications, hybridization strategies have also been investigated in the development of piperine-based derivatives. Xu et al. [20] synthesized a series of piperine-acylhydrazone hybrids, and compound 4o showed potent antiproliferative activity in A549 lung, HepG2 liver and HCT116 colon cancer cell lines. Taken together, piperine derivatives appear to have promising anticancer properties, although further studies are needed to validate their clinical relevance.

## Nanoformulations and Targeted Delivery of Piperine

Despite the potential therapeutic benefits of piperine, its clinical application remains challenging due to its hydrophobic structure and poor gastrointestinal absorption. Formulation approaches using nanotechnology has recently attracted interest as a promising approach to improve the pharmacokinetic behaviour and site-specific delivery of piperine through encapsulation in lipid-based systems, polymeric nanoparticles, nanomicelles and phytosomes [21, 22]. In addition, to enhance delivery efficiency, current formulations progressively incorporate targeting mechanisms, such as folic acid (FA) and hyaluronic acid (HA) conjugation, enabling the selective delivery of piperine to cancer cells overexpressing folate or CD44 receptors. The combination of nanoscale carriers with active targeting ligands represents a dual strategy that not only enhances intracellular uptake and accumulation at tumour sites, but also reduces off-target toxicity [23].

Piperine-loaded nanoemulsions were shown to enhance the cytotoxic effect of piperine against 4 T1 and MCF-7 breast cancer cells by improving its stability and release profile. This formulation showed potential as an effective delivery system for lipophilic anticancer agents by significantly lowering IC50 values [24]. In another study, various piperine derivatives were encapsulated into chitosan-based microgels to increase solubility and enable redox-responsive release, which led to enhanced anticancer activity against 4 T1 breast cancer cells even at low concentrations (100 µg/mL) [25]. Piperine was co-loaded with sorafenib into poly(lactic-co-glycolic acid) (PLGA) nanoparticles to increase the stability and therapeutic efficacy of the drug and resulted in improved cytotoxicity against HepG2 hepatocellular carcinoma cells compared to sorafenib alone [26]. Using a similar delivery strategy, another study demonstrated the incorporation of FA and HA ligands into PLGA-based redox-sensitive micelles, providing dual receptor targeting. The resulting DOX/FA-HA-SS-PLGA micelles showed improved cytotoxicity and apoptosis in breast (MCF-7), lung (A549) and colon (CT26) cancer cell lines, and enhanced antitumour efficacy and reduced systemic toxicity in vivo [27]. In conclusion, although preclinical studies of these nanoformulation and targeting strategies have shown promising results, further research is needed to validate their clinical applicability and therapeutic reliability.

## Mechanisms of Piperine’s Anticancer Effects

It is reported that piperine can show anticancer and antimutagenic activities against various cancer cells, cause apoptosis in cancer cells, and suppress metastasis and tumour development by affecting various proteins in apoptotic processes at the molecular level [8]. These mechanisms include the potential slowing effect of piperine on cancer formation and development by altering redox homeostasis. The reactive forms of procarcinogens, free radicals, and reactive oxygen species (ROS) produced as a result of metabolic activation are known to play key roles in the development of cancer. In this context, piperine may support cell death by affecting cellular physiology in redox homeostasis [28]. It may also affect essential transcriptional enzymes such as cyclins, cyclin–dependent kinases (CDKs), matrix metalloproteinase 9 (MMP-9), NF–κB, caspases, cyclic AMP–responsive element binding protein (CREB), and activated transcription factor- 2 (ATF-2). It may cause cell cycle arrest by downregulating the relevant mediators [15]. Additionally, piperine may have an inhibitory effect against angiogenesis. Thus, in addition to being involved in cell differentiation and autophagy processes, it may serve as a potential inhibitory agent against cancer development and progression by modulating various signalling pathways [29]. Furthermore, it has been documented that piperine can enhance the sensitivity of tumour cells to antineoplastic drugs [30]. These diverse molecular interactions suggest that piperine may represent a promising multi-targeted anticancer agent that offers potential advantages, such as the ability to interfere with key signaling pathways and affect multiple stages of cancer progression.

## Cell Cycle Arrest

Failure to regulate cell cycle control, a fundamental process responsible for the maintenance of appropriate cell proliferation and cellular integrity, is associated with cancer development. Cyclins, CDKs, and CDK inhibitors are protein groups responsible for controlling various cellular checkpoints [31]. It was shown that piperine significantly reduced the growth of HT-29 colon cancer cells by causing cell cycle arrest in the G1 phase and promoting apoptosis. This was accomplished via upregulating p21/WAF1 and p27/KIP1 and downregulating retinoblastoma protein phosphorylation, cyclins D1 and D3, and CDKs 4 and 6 [32]. Similarly, cell viability has been shown to significantly decrease with exposure to piperine at certain concentrations (25–300 µM) in human oral squamous carcinoma cells. Piperine has been found to trigger cell arrest in the G2/M stage and reduce DNA contents [33]. In another study, it was demonstrated that curcumin and piperine had antiproliferative potential in drug resistant human HL60 leukaemia cells. This was achieved by inducing apoptosis and autophagy, inhibiting cell migration, and arresting the cell cycle in the S phase [34]. The use of piperine, both alone and in conjunction with temozolomide, a chemotherapeutic agent employed in the clinical management of glioblastoma, has been demonstrated to facilitate apoptosis through the release of caspase–3, caspase–8, and caspase–9 together with the disruption of MMP activity and the suppression of wound healing and motility in vitro. The combined treatment significantly inhibited the expression of CDKs 2, 4, and 6, and this finding indicated a pause between the S phase and G1 [35]. Overall, the cell cycle regulators targeted by piperine exhibit a high degree of dependency on the specific type of cancer cell, resulting in variations in the stage of the cell cycle at which progression is blocked.

## Promotion of Apoptosis and Suppression of Cell Proliferation

Cancer pathogenesis is associated with the dysregulation of programmed cell death mechanisms including autophagy, necrosis, and apoptosis, the latter of which is the most basic cell death mechanism. The process of apoptosis is initiated by the proteolytic cleavage of a multitude of proteins, facilitated by the enzymatic activity of efficient caspases, including caspase-3, −6, and −7 [36]. Jafri et al. [37] demonstrated that exposure of human cervical adenocarcinoma cells to piperine in vitro resulted in significant dose dependent induction of apoptosis (25, 50, and 100 µM) and a concomitant suppression of the growth of the cancer cells accompanied by increased ROS production and delayed wound healing. In addition, by activating caspase – 3 and reducing the mitochondrial membrane potential, piperine promoted cell death in these cells. It was concluded that the growth suppression of cervical adenocarcinoma cells was associated with an arrest in the G2/M stage. In another study, radiotherapy–resistant HT–29 colorectal adenocarcinoma cells were pre- treated with piperine at 12.5 or 25 µg/mL prior to exposure to gamma radiation. The combined treatment was found to induce apoptosis in HT–29 cells in the G2/M phase to a larger extent than radiation therapy alone via the mitochondria dependent pathway. In the same study, the activation of caspase–3, a crucial molecule in apoptotic signalling, was markedly elevated, whereas the activation of oestrogen receptor beta (Erβ), a nuclear receptor transcription factor that facilitates tumour suppression, was enhanced with the application of piperine. Thus, it was concluded that piperine may increase cellular sensitivity to radiotherapy [38]. Similarly, the effects of piperine and doxorubicin (DOX), an anti – osteosarcoma drug, on U2OS and 143B osteosarcoma cells have been examined in both in vitro and in vivo conditions. The results demonstrated that cell proliferation and tumour growth were substantially inhibited in the combination therapy group compared to the monotherapy groups. An evaluation of apoptosis revealed that piperine upregulated the expressions of BAX and P53, which are proapoptotic proteins, and enhanced DOX–induced cell apoptosis by downregulating the expression of Bcl- 2 [39].

## Inhibition of Angiogenesis

While angiogenesis induces growth and metastasis by providing oxygen and nutrients to tumors, the metastasis process involves mechanisms such as decreased cell adhesion, increased cell invasion and motility, resistance to apoptosis, and proteolysis via MMPs. Piperine has been shown to reduce the production and expression of MMPs in a number of cancer cells [40]. Zare et al. [29] reported that piperine treatment effectively suppressed cell growth in MCF–7 breast carcinoma cells. Furthermore, the expression levels of VEGF and MMP–9, genes that play critical roles in angiogenesis, metabolism, and the invasiveness of breast cancer cells, were suppressed in a dose dependent manner with piperine treatment [5, 10, and 25 µM], while the expression of E – cadherin was induced. Other researchers reported that piperine could reduce neoplastic findings, including cell proliferation, survival, and migration, by inducing cell cycle arrest at the G1/G0 and G2/M stages in an in vitro model of cervical cancer and a normal cell line. It was suggested that piperine triggers apoptosis and inhibits angiogenesis by downregulating the COX– 2 pathway, which regulates the expression of MAPKs, MMPs, and their antagonists [41].

## Alteration of Redox Homeostasis

Oxidative stress causes irreversible damage to DNA, peroxidation of lipid cell membranes, and oxidation of proteins and inhibition of enzymes. Increased ROS levels under oxidative stress conditions lead to the activation of various oncogenic pathways [42]. Piperine can prevent cancer development via antioxidant activities at low doses (< 200 µg/mL) [14]. For example, the prophylactic piperine treatment of rats with induced colon cancer supported antioxidant responses such as HO1, GPx, GR, SOD, and CAT and reduced lipid peroxidation. Moreover, evidence indicates that it activates the nuclear factor erythroid 2 [Nrf 2] signalling pathway, which inhibits the NF–κB pathway and inflammatory mediators such as PGE2, TNF α, and IL 6. Consequently, it exhibits ROS scavenging activity [43]. Conversely, elevated doses of piperine have been observed to contribute to ROS production, which results in cell death in numerous types of cancer cells [44]. It is therefore thought that the pro – oxidative activity of piperine may be responsible for its ability to induce apoptosis in a variety of cancers [45]. It was established that cell proliferation was suppressed and apoptosis was induced dose dependently in human HGC27 gastric cancer cells treated with varying concentrations (10, 20, and 40 mg/L) of piperine. Piperine exerted those effects via the elevation of ROS and mitochondrial damage in cancer cells. In addition, piperine has been reported to increase the upregulation of key proteins involved in apoptosis, including Bcl2, BAX, and caspase 3 and 9 [46]. Buranrat et al. [47] stated that piperine induced apoptosis and inhibited colony formation in the KKU 100 and KKU M452 cholangiocarcinoma lines. Specifically, piperine treatment resulted in the cessation of the cell cycle at the G0/G1 stage in KKU 100 cells and at the S – G2/M stage in KKUM452 cells. The possible mechanism driving those findings was suggested to be linked to piperine decreasing the mitochondrial membrane potential and inducing ROS formation, similarly to the conclusions of Guo et al. [46]. Thus, the literature reveals that piperine has the capacity to act as both an antioxidant that prevents tumour formation and a pro oxidant that induces cancer cell apoptosis in a dose dependent manner.

## Inhibition of Cancer Stem Cells

**Fig. 1:**
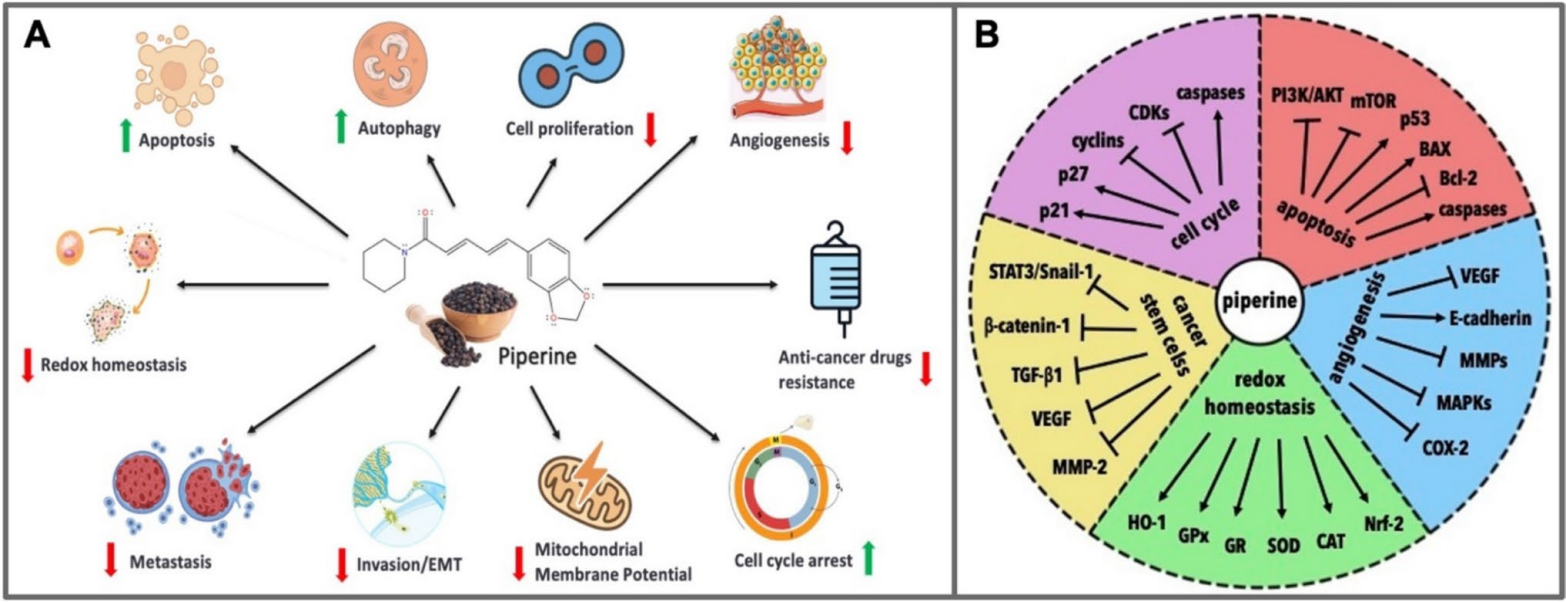
**A** Schematic illustration of piperine’s in vitro cellular effects, including the inhibition of cell proliferation, mitochondrial membrane potential (MMP), invasion/EMT, angiogenesis, redox homeostasis, metastasis, and drug resistance, as well as the stimulation of apoptosis, autophagy, and cell cycle arrest. **B** Overview of molecular pathways associated with these effects, highlighting the modulation of cell cycle regulators, apoptotic markers, angiogenic factors, redox enzymes, and cancer stem cell signaling. Closed arrows (→): Activation or increase. Blocked arrows (⊥): Inhibition or suppression

Cancer stem cells (CSCs) are a subtype of cancer cells with a distinct metastatic phenotype that play an important role in tumour initiation, metastasis, treatment resistance and recurrence [48]. It is therefore of therapeutic importance to facilitate the development of these cells into a more mature and metabolically quiescent phenotype. Recently published study have shown that low oxygen levels and the epithelial mesenchymal transition (EMT) pathway, entailing the transformation of epithelial cells into a more invasive and metastatic mesenchymal cell structure, may contribute to the persistence of CSCs [49]. Piperine’s interactions with these pathways and its triggering effects on CSC differentiation are being investigated. Song et al. [50] showed that piperine inhibited colon cancer metastasis, cell migration, and invasion through the EMT pathway mediated by STAT3/Snail in human SW480 and HCT – 116 colon cancer cells. In another study, piperine was found to be cytotoxic for A549 lung adenoma, MDA-MB-231 breast adenoma, and HepG2 hepatocarcinoma cells at concentrations above 100 µM and was also reported to reverse transforming growth factor beta (TGF–β1) induced EMT related processes [51]. Piperine has been shown to supress the expression of metastatic markers VEGF and MMP2 by blocking the Wnt/β catenin signalling pathway, which induces cell invasion and metastasis, in human 143B and U2OS osteosarcoma cells [52]. Additionally, piperine may have synergistic effects with other natural compounds in these signalling pathways. A recent study examined the cytotoxic action of piperine and curcumin against MCF-7 breast cancer cells and concluded that this combination suppressed cell viability, induced apoptosis, and arrested the cell cycle at certain stages. Furthermore, while EMT related genes such as Snail1, TGF- β1, and β catenin 1 are downregulated, the gene expression of tumour suppressor miRNAs has been shown to be upregulated [53]. The potential anticancer effects of piperine and the related molecular pathways are summarized in Fig. 1A–B.

## Piperine and Its Relationships With Certain Types of Cancer

### Breast Cancer

Several dietary compounds and alkaloids, including piperine, have proved beneficial in treating the occurrence or recurrence of breast cancer. Piperine is considered to be an effective chemopreventive agent in breast cancer through mechanisms of cell death and apoptosis, modulation of the levels of various signalling proteins, and inhibition of tumour growth [54]. Zhang et al. [55] reported that piperine triggered cell apoptosis in MCF-7 breast carcinoma cells by dose dependently increasing BAX expression and decreasing Bcl- 2 expression. In addition, by inhibiting the migration and invasion of MCF −7 cells, piperine inhibited the cell cycle at the G2 stage and suppressed breast cancer progression. It also reduced the ability of cancer cells to proliferate in obese mice by upregulating the miR-181c-3p pathway and downregulating PPARα in leptin induced breast cancer cells [56]. In another study, piperine, together with piperlongumine, another natural pepper alkaloid, suppressed the activation of STAT3 by working synergistically to counteract the uncontrolled growth of breast cancer cells and exerted a potent apoptosis inducing effect [57]. Breast cancer cells may be unresponsive to conventional anticancer drugs due to the presence of their carriers, such as drug – resistant P – glycoprotein, which is highly expressed on cell membranes. When piperine was administered alone or together with capsaicin to DOX–resistant cancer cells, a decrease in the levels of P-glycoprotein and breast cancer resistance protein (BCRP) was observed. This resulted in a decrease in DOX resistance in the cells, with these activities attributed to piperine independently [58]. Schmidt et al. [59] showed that piperine, curcumin, and resveratrol caused cytotoxic effects in MCF-7 cells and that these effects were associated with the inhibition of glyoxalase 1 activity via antiapoptotic properties as well as the decrease of mitochondrial membrane potential. Together with cisplatin, piperine reduced the expression of Bcl2, induced apoptosis by increasing the levels of caspase enzymes, and reduced the survival of human MCF7 breast cancer cells [60]. Additionally, the encapsulation of piperine in polymeric nanoparticles or liposomal formulations has been reported to yield synergistic effects with anti – breast cancer drugs. For instance, polyethylene glycol-conjugated liposomes containing tamoxifen and piperine showed improved cytotoxicity and increased cellular absorption in MCF-7 breast cancer cells, suggesting that this combination may improve treatment efficacy [61]. Its use in breast cancer treatment is promising as piperine may affect the development of breast cancer in various ways and new application methods continue to be developed.

### Gastrointestinal System Cancers

Although tobacco use and alcohol consumption are major risk factors for GI cancers and rare oral malignancies, the potential role of alkaloids such as piperine in the prevention and treatment of these cancers has gained importance in recent years. Oral malignancies are fairly infrequent and generally occur in people with long term histories of alcohol consumption and/or tobacco use [62]. Piperine triggers apoptosis and autophagy in vivo by blocking the PI3 K/AKT/mTOR signalling pathway in human HSC- 3 oral carcinoma [63]. The lipid matri –mediated nanoliposomal encapsulated form of piperine and quercetin exerted cytotoxic effects by inducing apoptosis in FaDu oral cancer cells in vitro [64]. Piperine was found to be a potent inhibitor of proliferation and an inducer of apoptosis in human SNU- 16 gastric cancer cells. More specifically, piperine showed antiproliferative effects by enhancing the upregulation of proapoptotic BAX, cytochrome c, PARP, and caspase-3 while downregulating the levels of Bcl- 2, Bcl—xl, and PI3 K/AKT [65]. Piperine showed selective cytotoxic activity in two different gastric cancer cell lines, including initial and metastatic cells. This indicates that its effectiveness increases in more aggressive cells. Additionally, when used together with traditional chemotherapeutic agents, it helped those drugs achieve the same cytotoxic effect at lower concentrations [66]. There are very few studies investigating the cancer – inhibiting properties of piperine in pancreatic cells. However, piperine was found to inhibit the cell cycle at stage G0/G1 in PANC-1 pancreatic cancer cells. It was reported to suppress cell proliferation, migration, and invasion by inhibiting the expression of cell cycle modulators such as FOXM1 and cyclin B1 and D1 [67]. The effects of piperine on GI tumours have been explored most thoroughly for colorectal cancer, one of the most prevalent human malignancies. Lithocholic acid is a bile acid thought to be involved in the invasion and migration of colon carcinoma cells. Piperine markedly inhibited angiogenesis by suppressing lithocholic acid induced IL8 expression in HCT116 colon cancer cells. This effect was shown to have occurred through the inhibition of ERK 1/2 and AKT via ROS signalling pathways [68]. In another study conducted on the HCT-116 cell line, administration of piperine alone did not directly inhibit cell proliferation. However, when used together with curcumin loaded emulsomes, piperine inhibited cellular proliferation by 50% by increasing the activity of curcumin. This combined treatment was also reported to inhibit cell cycle arrest at the G2/M stage and promote apoptosis by elevating caspase 3 levels [69]. It has been shown that the triple combination treatment of non – psychoactive cannabidiol/cannabigerol, piperine, and curcumin exerts synergistic antitumourigenic effects against the HCT 116 and HT29 colon adenocarcinoma cell lines by activating the Hippo YAP signalling pathway [70]. When used with the selective COX 2 inhibitor celecoxib [CXB], piperine increased the bioavailability of the drug in human HT29 colon adenocarcinoma cells and increased the concentration of CXB in the blood by 129% after oral administration. This combined treatment caused increased intracellular ROS, mitochondrial dysfunction, caspase activation, and apoptotic activity in cancer cells. It was also reported that CSC levels decreased with the modulation of the Wnt/β catenin signalling pathways [71].

### Gynaecological Cancers

Human papillomavirus (HPV) infection, which is most common in women, causes cervical cancer by reducing the activity of the p53 gene, which is an important contributor to apoptosis. In addition, the COX 2 enzyme can induce tumour formation, most notably in cervical cancer caused by ongoing HPV infection [72]. It has been shown that piperine treatment supports cell proliferation by increasing p53 expression and decreasing COX 2 levels in the HeLa cervical cancer cell line [73]. The piperine based P- glycoprotein inhibitor PGP 41 showed potent inhibitory action against the chemoresistant NCI/ADR RES ovarian cell line. Paclitaxel is one of the earliest medicines used to treat ovarian cancer and is also a P- glycoprotein metabolite. NCI/ADR- RES cells therefore have a high level of resistance to paclitaxel, but PGP – 41 increased the sensitivity of these cells to paclitaxel treatment. It was reported to be able to arrest cells in the G2/M phase, leading to the expression and destruction of apoptotic genes and cancer cells [74]. Moreover, the mechanism by which piperine increases paclitaxel sensitivity by downregulating phospho AKT and myeloid cell leukaemia 1, which has antiapoptotic functions, has also been demonstrated in resistant HeLa/PTX cervical cancer cells [75]. Piperine was found to suppress cell proliferation of the human A2780 ovarian cancer cell line by inducing apoptosis in a dose dependent manner at 8, 16, and 20 µM. This process occurred via the activation of mitochondrial cytochrome c, caspase 3 and 9, and PARP depletion. It was also reported that piperine showed anticancer potential via its actions in apoptotic pathways, reducing JNK and p38 MAPK phosphorylation in cancer cells. Alone or in combination with targeted anticancer drugs, piperine can decrease the drug resistance properties of ovarian cancer cells and show enhanced cytotoxic properties [76].

### Lung Cancer

The actions and underlying mechanisms of piperine against tobacco smoke – induced BEAS 2B and A549 lung cancer cells were investigated using in vitro and in vivo models. By modulating SIRT1, piperine reduced inflammatory biomarkers such as TNF α, IL 6, and IL–1β and promoted markers involved in the cell cycle and antioxidant systems such as Nrf2. As a result, piperine was reported to alleviate lung inflammation and inhibit EMT by modulating the inflammatory cascade [77]. Piperine has also been shown to reduce oncogene expression by stabilising the G quadruplex sequence in the promoter region of the proto oncogene c- myc in the A549 lung cancer cell line [78]. In another study involving A549 lung cancer cells, it was found that the use of piperine together with in vitro γ radiotherapy increased tumour apoptosis. However, further research is required to elucidate the molecular mechanisms underlying the radiosensitising effect of piperine [79].

### Prostate Cancer

Piperine has demonstrated the ability to inhibit prostate cancer cell proliferation and migration by targeting signalling pathways such as mTOR and MMP- 9 [80]. A recent study showed that piperine (15 µmol/mL) reduced cell viability by 50% in a model of PC- 3 prostate cancer. Additionally, it caused cell cycle arrest in the G0/G1 and G2/M stages. It was suggested that piperine achieved these effects by stimulating cell apoptosis and DNA fragmentation by targeting the AKT-1 protein [81]. Voltage gated K^+^ channels (KVs) have an important regulatory function in cancer cell proliferation and have been identified as potential targets for cancer therapy. Piperine has been shown to significantly block KVs in the LNCaP and PC-3 prostate cancer models in a dose dependent manner. Blocking KVs with piperine resulted in cell cycle arrest at the G0/G1 stage, the suppression of cell growth, and the dose – dependent induction of apoptosis [82]. Piperine and docetaxel, alone or in combination, were administered to ICR NOD/SCID mice with drug resistant prostate tumours, and compared to treatment with the drug alone, combined treatment with piperine exerted a significant tumour growth reducing effect by inhibiting P- glycoprotein activities. Additionally, intracellular concentrations of docetaxel were observed to increase when piperine was administered. Piperine may regulate important gene expression processes related to inflammatory reactions, angiogenesis, cell proliferation, and migration through the pathways described in prostate cancer [83]. Table 1 provides an overview of recent studies investigating the anti-cancer activity of piperine in both well-studied and less frequently explored cancer types.

**Table 1:** Recent studies evaluating the anti – cancer activity of piperine in different cancer types

Cancer type	Study design	Intervention		Main Findings	References
Breast cancer	In vitro and in vivo experimental study conducted on MDA-MB-231 triple-negative breast cancer cells and an Ehrlich ascites carcinoma mouse model	Piperine at concentrations of 100 and 200 μM to MDA-MB-231 cells for 72 h in vitro. In the in vivo model, piperine was administered intraperitoneally at a dose of 50 mg/kg daily for 15 days, combined with doxorubicin at 4 mg/kg weekly		Piperine enhanced the cytotoxic effects of doxorubicin on MDA-MB-231 cells, showing a synergistic interaction and reducing IC50 values of doxorubicin The combination treatment significantly suppressed PI3 K/Akt/mTOR signaling and upregulated PTEN expression, contributing to reduced CSC markers such as ALDH – 1 In the mouse model, the combination of piperine and doxorubicin reduced tumor volume, increased necrosis, and mitigated doxorubicin-induced cardiotoxicity	Hakeem et al*.* [84]
Lung cancer	In vitro and in vivo experimental study using iRGD-modified liposomes (LP) loaded with curcumin (CUR) and piperine (PIP) in A549 lung cancer cells and tumor-bearing nude mice	iRGD-LP-CUR-PIP was prepared and tested at concentrations ranging from 1.25 to 80 µg/mL in vitro, with incubation times of 24, 48, and 72 h. In vivo, 50 mg/kg iRGD-LP-CUR-PIP was administered intraperitoneally daily for 20 days, with comparisons to saline, LP-CUR, LP-CUR-PIP, and cisplatin		iRGD-LP-CUR-PIP demonstrated enhanced cellular uptake and cytotoxicity against A549 cells in a time and dose-dependent manner, with the most significant effects observed at 40 µg/mL after 48 h It significantly inhibited cell invasion and induced apoptosis, showing greater efficacy than LP-CUR and LP-CUR-PIP In the mouse model, iRGD-LP-CUR-PIP reduced tumor volume and weight, increased body weight and spleen index, and improved immune modulation compared to other treatments	Wang et al. [85]
Prostate cancer	In vitro study on DU145 and DU145/DTX prostate cancer cell lines to evaluate piperine's effects on docetaxel (DTX) sensitivity, apoptosis, and the Notch signaling pathway	Piperine at concentrations of 160 and 320 μM for 12, 24, 48, and 72 h. The combination of piperine with DTX was tested at DTX concentrations of 1, 5, 10, 25, 50, and 100 nM for 24 h		Piperine significantly enhanced the sensitivity of DU145 and DU145/DTX cells to DTX, reducing the IC50 value of DTX in a dose-dependent manner, with the most pronounced effect observed at 320 μM piperine combined with 10 nM DTX after 24 h Piperine induced apoptosis and inhibited migration and invasion of DU145 and DU145/DTX cells, with maximum apoptotic activity observed at 320 μM after 48 h Piperine downregulated Notch1, Jagged1, Hey1, and Hes1 expression in a concentration-dependent manner, particularly at 320 μM, highlighting its potential in overcoming DTX resistance	Wang et al. [86]
Colorectal cancer	In vitro and in vivo experimental study in HCT116, SW480 and CT26 colorectal cancer cells and xenograft mouse models	Piperine was applied to CRC cells at concentrations ranging from 50 to 150 μM for 24 to 48 h. In the xenograft mouse model, piperine was delivered orally at a dose of 20 mg/kg daily for 27 days		Piperine significantly inhibited cell viability and colony formation in CRC cells in a dose-dependent manner, with the highest inhibition observed at 150 μM after 48 h Piperine induced autophagy-dependent cell death by increasing ROS levels and inhibiting the AKT/mTOR signaling pathway In the xenograft model, piperine reduced tumor volume and weight, suppressed Ki67 expression, and enhanced LC3 expression, indicating autophagy activation	Xia et al. [87]
Gastric cancer	In vitro experimental study using the human gastric cancer cell line TMK – 1	Cells pretreated 0–100 µM piperine for 1 h prior to incubation with IL – 1β		Piperine inhibited IL – 1 β – induced IL – 6 expression in gastric cancer cells in a dose – dependent manner Piperine inhibited the activation of p38 MAPK and STAT3 signaling pathways and suppressed the invasiveness of gastric cancer cells	Xia et al. [88]
Ovarian cancer	In vitro*,* non – RC experimental study using human ovarian adenocarcinoma (SKOV – 3) and human keratinocyte (HaCaT) cell lines, with treatment groups of piperine alone, paclitaxel alone, and the combination of piperine and paclitaxel	Piperine at 10 μM and PTX at 5 nM, administered in combination		The combination of PTX and piperine showed a synergistic effect in inducing apoptosis in ovarian cancer SKOV – 3 cells The synergistic effect was mediated through the accumulation of ROS, leading to mitochondrial depolarization and DNA damage The treatment also caused cell cycle arrest in the G2/M phase, upregulation of pro – apoptotic BAX and caspase – 3, and downregulation of anti – apoptotic Bcl – 2	Pal et al. [89]
Cervical cancer	In vivo, non—RC animal study using a subcutaneous xenograft model in athymic nude mice. The mice were randomly divided into 4 groups: control, piperine alone, MMC alone, and the combination	Piperine, MMC, and the combination of piperine and MMC, incubated with Hela and Hela/MMC cells The participants received control, piperine (5 mg/kg), MMC (2 mg/kg), and the combination of piperine (5 mg/kg) and MMC (2 mg/kg) at 2 – day intervals for 4 weeks	The combination of piperine and MMC effectively overcame drug resistance in human cervical cancer cells The combination therapy suppressed the activation of STAT3 and NF – κB signaling pathways, leading to downregulation of the Bcl – 2 and upregulation of BAX inducing apoptosis The synergistic anti – tumor effects of piperine and MMC were demonstrated in a mouse xenograft model		Han et al. [90]
Leukemia	In vitro study on NB4 and MOLT-4 acute leukemia cell lines	Piperine at concentrations of 50, 100, and 200 μM for 24 and 48 h. The IC50 values were determined as 145 μM for NB4 cells and 156 μM for MOLT-4 cells after 48 h, which were used for subsequent experiments	Piperine significantly reduced cell viability in NB4 and MOLT-4 cells in a dose and time-dependent manner, with IC50 values achieved at 145 μM and 156 μM, respectively, after 48 h Piperine induced autophagy by upregulating ULK1, Beclin-1, and LC3 while suppressing mTOR and NF-κB1 expression Piperine enhanced senescence-associated β-galactosidase activity and increased p21 and IL-6 expression, while downregulating CDK2, promoting cell cycle arrest in leukemia cells		Charoensedtasin et al. [91]
Oral cancer	In vitro*,* non – RC experimental study was conducted to evaluate the anticancer effects of ZnO-PIP NPs on human oral squamous carcinoma KB cells	ZnO-PIP NPs administered at concentrations of 5, 25, 50, and 100 μg/mL for 24 h	ZnO-PIP NPs demonstrated a dose-dependent anticancer effect, significantly reducing KB cell viability at 100 μg/mL Apoptosis-related gene expression analysis showed downregulation of Bcl – 2 and upregulation of BAX and p53, promoting apoptosis		Shaik et al. [92]
Head and neck cancer	In vitro experimental study conducted on HEp-2 (laryngeal squamous cell carcinoma) and SCC-25 (tongue squamous cell carcinoma) cell lines	Piperine at concentrations of 25, 50, 100, 150, 200, 250, and 300 μM for 4, 24, 48, and 72 h The concentration of 150 μM and a treatment duration of 24 h were identified as the optimal conditions for the majority of the analyses	Piperine reduced cell viability and colony formation, induced apoptosis, and caused cell cycle arrest in the G2/M and S phases It also inhibited migration and invasion by modulating the expression of MMP2/9 and inflammatory markers (PTGS2, PTGER4, IL-8, and IFN-γ)		Gusson-Zanetoni et al. [93]
Melanoma	In vitro experimental study administered on B16 F10 mouse melanoma cells to assess the anti-tyrosinase and anti-melanogenic effects of piperine. Cells were treated with α-melanocyte stimulating hormone to induce melanogenesis	Piperine at concentrations of 11, 22, and 44 μM for 48 to 72 h. Cellular tyrosinase activity and melanin content were measured, with kojic acid (1407 μM) used as a positive control	Piperine at a concentration of 44 μM reduced cellular tyrosinase activity by 21.51 ± 2.00% without inducing cytotoxicity At the same concentration, it significantly lowered intracellular melanin content by 37.52 ± 2.53% by downregulating the transcription of tyrosinase and TRP-1, while leaving TRP-2 unaffected Piperine demonstrated a promising potential as a depigmenting agent due to its efficacy at lower, non-toxic concentrations		Khongkarat et al. [94]
Hepatocellular carcinoma	In vitro experimental study on HepG2, Hep3B, and non-cancerous AML12 cell lines to evaluate the effects of piperine on cellular stresses, apoptosis, and cytotoxicity, as well as its interaction with sorafenib	Piperine at concentrations of 25, 50, 75, 100, 125, 150, 200, and 250 μM for 48 h. In combination studies, IC50/4, IC50/2, and IC50 concentrations of piperine were tested with sorafenib concentrations ranging from 0.5 to 10 μM	Piperine induced cellular stresses and apoptosis in hepatocellular carcinoma cells in a concentration-dependent manner, activating the JNK signaling pathway Pre-treatment with a JNK inhibitor significantly reduced piperine-induced cytotoxicity, cellular stresses, and apoptosis, demonstrating JNK's critical role in piperine's effects Piperine displayed additive or synergistic effects with sorafenib in hepatocellular carcinoma cells, as evidenced by combination index values ≤ 0.9		Sayilan Ozgun et al. [95]

AKT, Protein kinase B; ALDH – 1, Aldehyde dehydrogenase 1; BAX, Bcl-2-associated X protein; Bcl – 2, B-cell lymphoma gene-2; CDK2, Cyclin-dependent kinase 2; CSC, Cancer stem cells; GPx, Glutathione peroxidase; IC50, Inhibitory concentration 50%; IFN-γ, Interferon-gamma; IL—8, Interleukin – 8; iRGD, Internalizing arginine-glycine-aspartic acid peptide; JNK, c-Jun N-terminal kinase; LC3, Microtubule-associated protein 1 A/1B-light chain 3; MAPK, Mitogen-Activated protein kinase; MDM-2, Mouse double minute 2; MMC, Mitomycin-C; MMP, Matrix metalloproteinase; mTOR, Mammalian target of rapamycin; NF-κB, Nuclear factor kappa B; PARP-1, Poly ADP-ribose polymerase 1; PI3 K, Phosphatidylinositol 3-kinase; PTEN, Phosphatase and tensin homolog; PTGER4, Prostaglandin E receptor 4; PTGS2, Prostaglandin – endoperoxide synthase 2; PTX, Paclitaxel; RC, Randomized controlled; STAT-3, Signal transducer and activator of transcription 3; TIMP-1, Tissue inhibitor of metalloproteinases-1; TNBC, Triple-negative breast cancer; TRP, Tyrosinase-related proteins; ULK1, Unc-51-like autophagy activating kinase 1; ZnO-PIP NPs, Piperine-coated zinc oxide nanoparticles

### Safety, Toxicity, and Pharmacological Limitations of Piperine

Despite the promising therapeutic benefits of piperine, its application in clinical practice is limited due to concerns about safety and pharmacokinetics. Piperine has shown acute toxicity in various animal models. The LD₅₀ values have been reported to vary between 15.1 and 514 mg/kg, depending on the administration method. Adverse effects, including respiratory paralysis, gastrointestinal and adrenal tissue damage, have been observed in high-dose administration cases [96]. In human intervention studies, piperine has been administered alone or in combination, usually in doses ranging from 4–40 mg/day and for periods ranging from 4 days to 6 months. Nevertheless, data on adverse effects are quite limited. Only a few studies have observed mild gastrointestinal adverse effects (*e.g*., abdominal discomfort, diarrhea) or, rarely, skin rash. However, due to the insufficient safety data available, caution is advised regarding the bolus intake of isolated piperine [15, 97]. In addition, in a 90-day subchronic oral toxicity study, piperine was administered to rats at doses of 5, 15 and 35 mg/kg/day; no histopathological or hematological toxicity was observed and the researchers determined these doses to be safe. In the same study, although the European Food Safety Authority (EFSA) defined the NOAEL dose as 5 mg/kg/day based on an increase in serum cholesterol, the study stated that this increase was not toxicologically significant, and doses up to 50 mg/kg/day were reported to be safe [98, 99]. Beyond toxicity and safety concerns, another key pharmacological limitation of piperine is its strong potential to interact with common drugs through modulation of critical metabolic pathways. Piperine has significant effects on various drug metabolizing enzymes and transporter proteins. It has been shown to increase drug bioavailability by inhibiting cytochrome P450 enzymes (especially CYP3 A4) and P-glycoprotein [100]. A recent meta-analysis reported significant increases in peak plasma concentration (Cmax), area under the curve (AUC), and half-life of conventional drugs administered with piperine. The findings suggest that piperine may increase the bioavailability of drugs by inhibiting key enzymes such as CYP2 C9, CYP2E1, and CYP3 A4. This has clinical potential, especially in increasing the efficacy of drugs with poor bioavailability [101]. However, it has been found that piperine administration at a dose of 20 mg/day for 7 days caused a 31–59% increase in the AUC values ​​of drugs that are CYP3 A4 substrates, such as simvastatin, ritonavir, nifedipine, and cyclosporine. These results indicate potential risks associated with the concomitant use of piperine and narrow therapeutic index drugs [102]. This interaction profile observed with conventional drugs presents both opportunities and risks in the use of piperine in cancer treatment [100]. It has been reported that piperine suppresses cell proliferation, induces apoptosis and reduces drug resistance when used together with chemotherapeutic agents such as docetaxel, paclitaxel, cisplatin and 5-fluorouracil. This synergistic effect can both increase treatment efficacy and reduce chemotherapy-related side effects. However, these data are mostly based on in vitro and animal studies [103]. Clinical studies of piperine on cancer patients have not yet been conducted [100]. Therefore, the efficacy and safety profile of piperine-based combination therapies require critical evaluation, and future well-designed clinical trials are needed to validate these findings.

## Conclusions and Future Perspectives

Today, in addition to the current treatment methods for cancer, such as surgery, radiotherapy and chemotherapy, interest in alternative strategies is also increasing. These strategies arise from needs such as reducing the side effects of patients'treatment processes, preventing the development of resistance to treatment and improving the quality of life. It is conceivable that natural compounds, especially phytochemicals such as piperine, may have significant potential effects in cancer treatment and prevention in recent years.

This review sheds light on piperine's potential anticancer mechanisms and its effects on some types of cancer. Piperine, in addition to its various therapeutic properties that indirectly affect cancer, such as antiinflammatory, antioxidant and immunomodulator, also has properties that directly induce apoptosis in cancer cells, regulate cell cycle, inhibit metastasis and angiogenesis. In addition, piperine may serve as a promising natural adjuvant to conventional cancer therapies by potentially improving drug bioavailability and reducing the development of resistance.

Today, the majority of studies investigating the anticancer effects of piperine are based on in vitro cell studies. Given the limited number of existing human studies, more comprehensive and numerous studies are needed to better elucidate the specific molecular targets and pathways affected by piperine. These studies should evaluate its efficacy and safety in various types of cancer, as well as investigate its use at different doses and in bioavailability enhanced formulations, or in combination therapies with various phytochemicals and anti cancer drugs.

From a dietary point of view, the incorporation of spices, especially black pepper, which is a major source of piperine, in a balanced and adequate diet may provide general health benefits due to its antioxidant and anti-inflammatory properties. However, current evidence directly relating black pepper consumption to a reduced risk of cancer is insufficient given the lack of epidemiological and clinical studies to establish this relationship. Furthermore, although piperine has been studied in clinical trials for a number of other non-cancer conditions, such as osteoarthritis, fatty liver disease, and diabetes, no clinical trials to date have evaluated its potential effects in cancer patients. Therefore, future research is crucial to better understand the possible anti-cancer effects of piperine-rich diets and to provide a scientific basis for their use. Rather than using these compounds as supplements, it may be more beneficial to obtain them from natural food sources that are rich in and/or fortified with these compounds.

## Limitations

This review is limited by the lack of clinical studies evaluating the anticancer effects of piperine, since most of the available data are derived from in vitro or preclinical models. Although nanoformulations and combined therapies with piperine appear promising, their clinical efficacy, pharmacokinetics, and long-term safety have not been fully established. Also, this review may be subject to publication bias, as studies with positive findings are more likely to be published and included. For this review, an attempt was made to primarily include studies published within the past five years to ensure the inclusion of current and relevant findings. Finally, to maintain scientific integrity and data reliability, only articles indexed in well-established databases such as PubMed and Web of Science were considered. Studies that were not peer-reviewed or published in sources not indexed in these databases were excluded from the analysis.

## Data Availability

No datasets were generated or analysed during the current study.

## Abbreviations

AKT: Protein kinase B
ATF-2: Activated transcription factor 2
BAX: Bcl – 2 associated X protein
Bcl-2: B – cell lymphoma gene – 2
BCRP: Breast cancer resistance protein
CAT: Catalase
CDK: Cyclin dependent kinases
COX: Cyclooxygenase
CSC: Cancer stem cells
CXB: Celecocipitin
DOX: Doxorubicin
EFSA: European Food Safety Authority
EMT: Epithelial – mesenchymal transition
Erβ: Estrogen receptor beta
ERK: Extracellular signal regulating kinase
FOX: Forkhead box
GI: Gastrointestinal
GPx: Glutathione peroxidase
GR: Glutathione reductase
GSK3β: Glycogen synthase 3 – kinase beta
HO-1: Heme oxygenase – 1
IARC: International Agency for Research on Cancer
IL-6: Interleukin 6
JNK: C – Jun N – terminal kinase
KV: Voltage gated K + channels
MAPK: Mitogen – Activated protein kinase
Mcl – 1: Myeloid cell leukemia
MMP: Matrix metalloproteinase
mTOR: Mammalian target of Rapamycin protein complex
NF: κB, Nuclear factor kappa B
NOAEL: No Observed Effect Level
Nrf – 2: Nuclear factor erythroid 2
PARP: Poly ADP – ribose polymerase
PGE2: Prostaglandin E2
PI3k: Phosphatidylinositol – 3 kinase
PPAR: Peroxisome proliferator activated receptor
ROS: Reactive oxygen species
SOD: Superoxide dismutase
STAT – 3: Signal transducer and activator of transcription 3
TGF – β1: Transforming growth factor beta
TNF – α: Tumour necrosis factor alpha
VEGF: Vascular endothelial growth facto
